# Immune Exhaustion: Past Lessons and New Insights from Lymphocytic Choriomeningitis Virus

**DOI:** 10.3390/v11020156

**Published:** 2019-02-13

**Authors:** Shannon M. Kahan, Allan J. Zajac

**Affiliations:** Department of Microbiology, University of Alabama at Birmingham, Birmingham, AL 35294, USA; skahan@uab.edu

**Keywords:** LCMV, immune exhaustion, T cells, checkpoint inhibitors, chronic infection, antiviral responses

## Abstract

Lymphocytic choriomeningitis virus (LCMV) is a paradigm-forming experimental system with a remarkable track record of contributing to the discovery of many of the fundamental concepts of modern immunology. The ability of LCMV to establish a chronic infection in immunocompetent adult mice was instrumental for identifying T cell exhaustion and this system has been invaluable for uncovering the complexity, regulators, and consequences of this state. These findings have been directly relevant for understanding why ineffective T cell responses commonly arise during many chronic infections including HIV and HCV, as well as during tumor outgrowth. The principal feature of exhausted T cells is the inability to elaborate the array of effector functions necessary to contain the underlying infection or tumor. Using LCMV to determine how to prevent and reverse T cell exhaustion has highlighted the potential of checkpoint blockade therapies, most notably PD-1 inhibition strategies, for improving cellular immunity under conditions of antigen persistence. Here, we discuss the discovery, properties, and regulators of exhausted T cells and highlight how LCMV has been at the forefront of advancing our understanding of these ineffective responses.

## 1. Introduction

### Lymphocytic Choriomeningitis Virus (LCMV) and the Definition of T Cell Exhaustion

Since its discovery in the mid-1930s [1,2] lymphocytic choriomeningitis virus (LCMV) has become a proven system for defining fundamental immunological concepts. The ability of LCMV to establish both acute and chronic infections together with the primacy of the T cell response for mediating viral clearance and also causing lethal immunopathology, coupled with the power of mouse genetics, has enabled LCMV to be at the forefront of immunological research for decades. Although the study of LCMV in mice has an impressive track record of advancing our understanding of immunity, it is a natural mouse pathogen; therefore, the general applicability of any of the findings to humans and other pathogens requires verification. Nevertheless, a central strength of the LCMV system is that many of the discoveries regarding both cellular and humoral immunity have indeed been confirmed in humans. These common immunological themes include the formation of effector and memory T cell subsets, the longevity of adaptive immunity, and the development of T cell exhaustion. Most notably, these advances have helped underscore the development of approaches to improve human health, including the design of anti-cancer checkpoint blockade therapies. The importance of LCMV is evidenced by its roles in the award of the 1996 Nobel Prize for Medicine for the discovery of major histocompatibility complex (MHC)-restriction and the 1960 Nobel Prize for Medicine for the discovery of immune tolerance [3,4].

The ability of LCMV to establish lifelong persistence following in utero or neonatal infection, resulting from the presence of viral antigen in the thymus, provided foundational insights into the mechanisms of central tolerance. Importantly, although LCMV infection results in a viral carrier state following neonatal exposure, certain isolates of LCMV also cause a chronic infection in immunocompetent adult mice. Viral persistence in adult mice is, however, not always the case. Intracranial administration of LCMV can cause an immune-mediated lethal infection. In addition, intravenous or intraperitoneal injection of certain commonly used strains of LCMV leads to a self-limiting, fully resolved, acute infection and this approach is widely used to study effector T cell formation and immunological memory.

The ability of LCMV to establish chronic infections was critical for the discovery of T cell exhaustion and this is governed by its tropism and is also influenced by the viral replication rates [5,6,7,8,9,10,11]. In the case of the commonly used LCMV Clone 13 strain, these parameters are dictated by two amino acid residues, one at position 260 of the viral glycoprotein (GP260) and another within the polymerase (L1079) [6,9,10,11,12,13]. A third asparagine to aspartic acid mutation has also been identified (GP176) but is unnecessary for viral persistence and immune exhaustion [6,11]. The importance of the GP260 and L1079 residues is further illustrated by analyses of viral variants isolated from immunodeficient mice in which the parental acute Armstrong strain can establish a chronic infection due to the disabled immune response. In these instances, the recovered persisting viral variants generally have mutations at the L1079 and especially GP260 positions demonstrating that viral evolution can promote the establishment of a chronic infection [10].

By comparison with the parental acute (Armstrong) strain the lysine to glutamine L1079 mutation in the chronic LCMV Clone 13 isolate allows the virus to replicate more rapidly, increasing the levels of antigen. Strains carrying the Clone 13 variation at L1079 induce higher and longer lasting viremia with more viral RNA detectable in the spleen and liver [6]. These variants elicit more pronounced loss of T cell functions demonstrating that the rate of viral replication and subsequent increase in the viral loads promote T cell exhaustion. The GP260 phenylalanine to leucine mutation also contributes to viral persistence by altering viral tropism [11]. The LCMV Clone 13 variant of GP260 (leucine) allows the virus to attach more efficiently than the acute Armstrong strain (GP260 phenylalanine) to the cellular receptor alpha-dystroglycan, permitting more efficient infection of a broader range of cells including dendritic cells and macrophages as well as non-hematopoietic stromal cells. LCMV strains with high affinity for alpha-dystroglycan can, therefore, rapidly infect a large number of cells due to their expanded tropism and limit the host’s immune response, thus favoring persistence [10,11,14,15]. The resulting infection of dendritic cells also impairs their development and maturation, lowers their expression of MHC I and II, and decreases the induction of costimulatory molecules, including CD40 and CD80, thus weakening their ability to efficiently present antigen and fully activate anti-viral T cells [14,16,17,18].

Both GP260 and L1079 mutations facilitate LCMV Clone 13 infection of non-hematopoietic fibroblastic reticular cells (FRC) [8,19,20]. FRCs provide an immune scaffold and facilitate the delivery of chemokines such as CCL19 and CCL21 which recruit T cells and dendritic cells to the T cell zones within lymphoid organs, thus enhancing their interactions. Disturbing this coordination leads to dysfunctional interactions between T cells and dendritic cells and ineffectual priming of the T cell response [19,21]. FRCs also deliver support in the form of IL-7, a cytokine that provides survival signals to T cells. Interestingly, administering exogenous IL-7 during chronic LCMV infection aids the recovery of exhausted CD8 T cell responses [22,23]. Notable disruptions in lymphoid structure and FRCs also occur during human immunodeficiency virus (HIV) infection [24,25], further illustrating how the use of LCMV can advance our understanding of the outcomes of other persistent infections.

The earliest descriptions of immune exhaustion were derived from studies focusing on why anti-viral CD8 T cells in immunocompetent mice can eradicate acute but not chronic LCMV variants despite the viruses expressing identical T and B cell epitopes. The first studies revealed that there is a dearth of virus-specific CD8 CTL activity in chronically infected mice and showed the deletion of transferred virus-specific cells after high dose infection with the LCMV-docile strain, which induces a chronic infection in wild-type adult mice [26]. The development of MHC tetramers permitted the tracking of the natural endogenous CD8 T cell response to LCMV over time without relying on T cell receptor (TCR) transgenic systems or functional readouts. Using MHC tetramer technology two reports demonstrated that during chronic LCMV infections anti-viral CD8 T cell responses were elicited and did not necessarily become deleted following priming [27,28]. Instead, anti-viral CD8 T cells persisted for prolonged periods in chronically infected hosts but lost their full array of effector functions necessary to resolve the infection, resulting in the development of exhaustion.

Exhaustion is distinct from other dysfunctional states such as anergy, which arises after incomplete priming of naïve T cells during their interactions with antigen presenting cells (APCs); central tolerance, such as that arising in neonatally LCMV-infected carrier mice which results in thymic deletion of virus-specific T cells; and peripheral tolerance where T cell activation is repressed against perceived self-antigens as a mechanism to prevent immune pathology [29]. Exhaustion, however, has plausibly evolved to benefit the host by extinguishing an overly vigorous and potentially pathogenic response while permitting some limited level of ongoing immune surveillance.

While the earliest evidence of immune exhaustion came from studies using LCMV infection of mice, it has subsequently been found to commonly occur during numerous other chronic viral infections including HIV, hepatitis B virus (HBV), and hepatitis C virus (HCV) as well as non-viral infections such as malaria and tuberculosis [30,31,32,33,34]. Exhaustion also occurs in non-infectious environments, such as tumors, where tumor antigens are persistently expressed [35,36,37]. Thus, although many of the key concepts about immune exhaustion have been gleaned from studies using LCMV, they have been shown to be directly relevant to infections and pathologies in humans and have provided an informative foundation for better understanding immune mediated control in situations where the priming antigen persists.

## 2. The Properties and Molecular Regulators of Exhausted T Cells

### 2.1. Loss of Function

**Figure 1:**
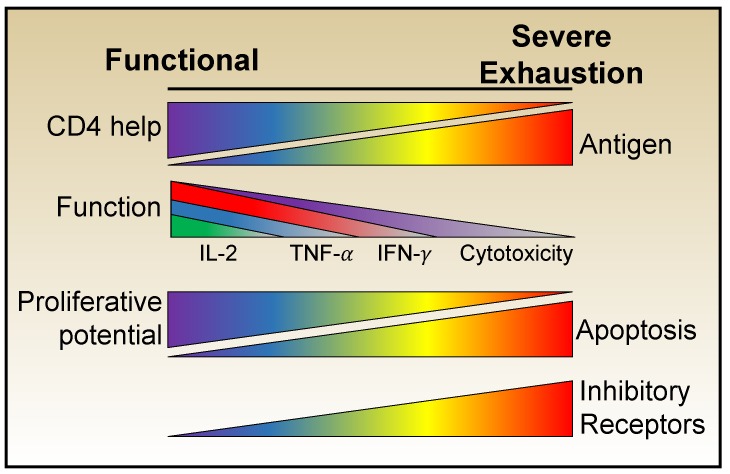
CD8 T cell exhaustion develops in a progressive manner as cardinal T cell effector functions are lost. Ineffective CD4 T cell help and sustained antigenic activation lead to the erosion of effector activities and can ultimately result in apoptosis. The severity of CD8 T cell exhaustion is reflected and exacerbated by the expression of multiple inhibitory receptors which further diminish functional capabilities.

The cardinal trait of exhausted T cells is their loss of functionality. Studies comparing anti-viral CD8 T cell development during acute and chronic LCMV infections have clearly shown that, at the population level, a progressive and predictable erosion of effector potential occurs over time in the chronically infected host. At the earliest stages the differentiation of prototypic effector cells is corrupted, with the loss of IL-2 production and then TNF-α synthesis becoming apparent [38,39,40]. Sustained antigenic activation together with an unfavorable cytokine milieu and inferior cellular support result in the further deterioration of functional potential signified by the loss of the ability to produce IFN-γ. Cytolytic activities are more resistant to exhaustion, which may allow the anti-viral population to continue to contribute to viral control [38,41,42], a concept that has been demonstrated by surges in viral loads after the deliberate deletion of CD8 T cells during chronic simian immunodeficiency virus (SIV) infection [43,44]. Moreover, exhausted T cells also lose their responsiveness to inflammatory cytokines which usually induce activation and effector cytokine synthesis by conventional effector and memory cells [45]. Thus, a spectrum of exhausted states can emerge, which vary in their severity of functional defects (Figure 1).

Over the past 20 years it has become apparent that exhausted cells are heterogeneous and exhibit a variety of phenotypes with distinct propensities for proliferation, survival, and self-renewal. Technological advances and the experimental amenability of the LCMV system have allowed more in-depth insights into the transcriptional, metabolic, and epigenetic features that refine the definition of exhaustion beyond that of simply a dysfunctional cell to include, more precisely, a heterogeneous state that compromises between viral control and damaging immunopathology.

### 2.2. Inhibitory Receptors

One of the defining features of exhausted T cells is their sustained high expression of portfolios of inhibitory receptors including PD-1 (CD279), CTLA-4 (CD152), LAG-3 (CD223), Tim-3, 2B4 (CD244), CD160, and TIGIT [46,47,48,49,50,51,52,53,54]. These receptors are typically transiently upregulated by CD8 T cells following stimulation during acute infections, where they play a role in attenuating the activation of the response. During chronic infections, the sustained high expression of inhibitory receptors constrains T cell functions and compromises viral control. As exhaustion progresses the number and levels of inhibitory receptor expression increases and contributes to the heterogeneity of the exhausted T cell pool. The identification of the role of inhibitory receptors in regulating exhausted LCMV-specific responses has proven critical for understanding the mechanisms of exhaustion, uncovering potential targets for bolstering inferior cellular immune responses, and fueling the explosive interest in checkpoint blockade therapies.

The most well studied exhaustion-associated inhibitory receptor is Programmed Cell Death-1 (PD-1). While it is not expressed on naïve T cells, PD-1 is transiently expressed after activation and functions to down-modulate the anti-viral response [55,56]. However, during chronic LCMV infection the levels of PD-1 remain elevated on CD8 T cells as exhaustion sets in [46,47,57,58,59]. High levels of PD-1 expression is a common feature of T cells during other chronic infections including HIV, HBV, and HCV [30,60,61,62]. PD-1 is also expressed by tumor-reactive T cells during many cancers, and targeting this inhibitory pathway is the basis of a major checkpoint blockade approach for cancer therapy [35,36,63,64,65,66].

PD-1 signals influence multiple T cell functions including TCR signaling, co-stimulation, motility, proliferation, and metabolism. PD-1 is a transmembrane protein in the B7:CD28 family of receptors that contains carboxy-terminal intracellular immunoreceptor tyrosine-based inhibition motifs (ITIMs) and immunoreceptor tyrosine-based switch motifs (ITSMs). Mechanistically, PD-1 interacts with its ligands, PD-L1 and PD-L2, resulting in recruitment of SHP1 and SHP2 to its ITAM and ITSM sequences which inhibits the phosphorylation of ZAP70 curtailing TCR signaling. SHP2 also interferes with PKCθ which inhibits CD28 signaling [67,68]. Furthermore, PD-1 signals also induce the E3 ubiquitin ligase Cbl-b which negatively impacts the surface expression of the TCR and thereby raises the threshold for antigen recognition [69,70]. In addition to increasing the amount of TCR signaling required for activation and the development of effector functions, PD-1 also suppress the immune response by reducing the duration of APC:T cell interactions [71]. Moreover, ligation of PD-1 modulates cellular metabolism by inhibiting PI3K and the mTOR pathway which curbs glycolysis and shifts cells toward oxidative phosphorylation [72,73,74,75]. Thus, PD-1 promotes exhaustion through its ability to negatively regulate multiple T cell activities.

Given the presence of sub-functional T cells during chronic viral infections, an important objective has been to restore their effector functions and improve infection or tumor control. Seminal studies demonstrated that antibodies which block PD-1 signals revitalize exhausted CD8 T cells during chronic LCMV infection [46]. Blockade of PD-1 signals can partially restore motility, proliferation, and the metabolism of exhausted CD8 T cells [46,48,49,57,72,75,76,77]. Additionally, these rescued cells exhibit greater IFN-γ and TNF-α production and increased cytotoxic potential when compared to virus-specific CD8 T cells in untreated mice. Consequently, PD-1 blockade therapies elevate the total numbers of functional virus-specific CD8 T cells and lower viral loads. Since these initial findings, PD-1 checkpoint blockade has been successfully applied to treat numerous types of cancers in humans [78,79,80,81,82]. The development of PD-1 blockade as a therapy for chronic viral infections in humans has not advanced as quickly, but the efficacy of PD-1 blockades have been demonstrated in other chronic viral systems including SIV and HCV in non-human primates [60,61,83,84]. 

During chronic LCMV infection, exhausted T cells express several other inhibitory molecules in addition to PD-1, including Tim-3, LAG-3, CD160, and 2B4. Nevertheless, the impact these molecules have on T cell function and the prospect for these molecules as targets for immunotherapy is less established. During acute LCMV infection Tim-3 is transiently upregulated on CD8 T cells, but expression remains elevated during chronic LCMV infection. Tim-3 co-expression with PD-1 identifies populations of CD8 T cells that are more exhausted and less functional than their PD-1^+^ Tim-3^-^ counterparts [49,85]. Tim-3 expression is dispensable for exhaustion, however, Tim-3 expression impacts T cell revitalization during PD-1 blockade [85]. Interest in LAG-3 as a regulator of T cell exhaustion grew after it was found to be expressed at elevated levels on CD8 T cells during chronic LCMV infection and this was heavily associated with co-expression of PD-1 [47]. However, despite its role as a negative regulator blocking LAG-3 alone fails to rescue exhausted T cells or accelerate viral clearance [47,52]. Levels of T cell exhaustion and viral clearance were also similar in wild-type and LAG-3 deficient mice during chronic LCMV infection [52]. CD160 and 2B4 are also elevated on anti-viral T cells during chronic LCMV infection, implicating their potential role in controlling exhaustion [47].

The success of targeting PD-1 pathways for improving inferior responses has led to other inhibitory receptors being validated as blockade therapies with varying results. Blockade of Tim-3 alone has a minimal effect on the recovery of the CD8 T cell response; however, co-blockade of PD-1 and Tim-3 is more effective than inhibiting Tim-3 and PD-1 individually [49]. Additionally, co-blockade of PD-1 and LAG-3 acts synergistically to resurrect exhausted cells and aid viral clearance [47] PD-1 blockade has also been combined with other immunotherapies such as IL-2 and IL-7 treatments, the depletion of regulatory T cells (Tregs), and agonism of costimulatory pathways leading to the synergistic boosting of exhausted T cells [76,86,87].

Collectively these findings, shown using LCMV, demonstrate the mechanistic roles of inhibitory receptors in perpetuating T cell exhaustion. They also reveal the non-redundant activities of these inhibitory receptors that can be targeted separately or in combination to improve the control of not only LCMV but also other viral infections and tumors.

### 2.3. Transcriptional Regulators

Genomic analyses of LCMV-specific CD8 T cells isolated from acute and chronic infections have revealed that exhaustion is regulated at the transcriptional, translational, and epigenetic levels [88,89,90,91]. While many transcriptional properties are shared between acutely activated and exhausted LCMV-specific T cells, global transcriptome analyses have uncovered distinct features of exhausted cells [59,89]. Like effector cells, exhausted CD8 T cells express low levels of memory-associated transcripts such as *ccr7, sell*, and *il7r* [59], and they generally also express fewer transcripts associated with resting naïve or memory T cells [89]. As expected, exhausted cells do express higher levels of transcripts encoding inhibitory receptors. There are also substantial transcription-associated differences between effector and exhausted cells in pathways related to cellular signaling, migration, survival, and metabolism. Thus, exhausted cells are transcriptionally distinct from both prototypic effector and memory subsets.

Exhausted CD8 T cells continue to express transcripts for certain effector genes such as *ifng*, despite poor protein expression and low functionality [59,88,92]. This implicates the significance of post-transcriptional regulation in controlling protein levels and the effector repertoire of anti-viral T cells. The failure of exhausted T cells to manufacture IFN-γ, despite the presence of transcripts, is likely a result of curtailed translation because of ineffective recruitment to ribosomes due to the suppressive factor, ZFP36L2, binding to 3’ AU-rich elements in the mRNA [93].

A single master transcription factor that determines exhaustion has not been identified. Instead the combined actions of numerous transcriptional regulators, including T-bet, Eomes, NFAT, Blimp-1, BATF, IRF-4, TCF-1, VHL, ID2, ID3, FOXO1, and TOX collectively contribute to the gradation of exhausted phenotypes [41,75,89,94,95,96,97,98,99,100,101,102]. The precise actions of these factors are dependent on context and can differ during acute and chronic infections, as well as vary depending on the developmental stage of the responding T cell. Along with changes in expression levels, variances in chromatin accessibility, DNA methylation, localization, and the ratio of binding partners can contribute to shifts in how transcription factors act in exhausted cells. During acute LCMV infection NFATc1 and Blimp-1 are positive and negative regulators of PD-1, respectively, but the roles of these transcription factors may change during chronic LCMV infection [103,104]. In exhausted cells, the levels of Blimp-1 and PD-1 are elevated, with higher PD-1 levels directly correlating with Blimp-1 levels. Moreover, the ablation of Blimp-1 leads to decreased levels of PD-1 demonstrating that in exhausted cells Blimp-1 is not a repressor of PD-1 [105]. Additionally, the nuclear translocation of NFATc1, which drives the expression of PD-1 during acute infection has been reported to be reduced in exhausted cells, suggesting that NFATc1 is not a primary inducer of PD-1 [41]. Nevertheless, contrary observations have demonstrated that NFAT expression is a driver of exhaustion due to changes in its interaction with the transcription factor AP-1. These discordances possibly reflect system or isoform-specific differences in the requirements for this transcriptional regulator [98].

During chronic LCMV infection PD-1 expression is induced by the transcription factor FOXO1, which steers the development of terminally exhausted PD-1^hi^ Eomes^hi^ CD8 T cells [75]. The transcription factors T-bet and Eomes also influence distinct transcriptional networks during acute and chronic infections [89]. Eomes is typically associated with memory CD8 T cells following acute LCMV infection, but during chronic LCMV infection Eomes is linked to terminally exhausted cells with poor survival and proliferative abilities [89,99]. Conversely, T-bet, which is associated with terminal effector differentiation during acute infection, is linked to less exhausted, stem-like progenitor CD8 T cells during chronic infections. This is achieved in part by the ability of T-bet to restrict full expression of PD-1 and is consistent with exhausted T-bet^hi^ PD-1^int^ CD8 T cells being more sustainable and susceptible to checkpoint blockade treatments [106]. Together these findings demonstrate that transcription factor networks play context-dependent roles in regulating exhausted cells which are distinct from those in their naïve, effector, and memory counterparts.

**Figure 2:**
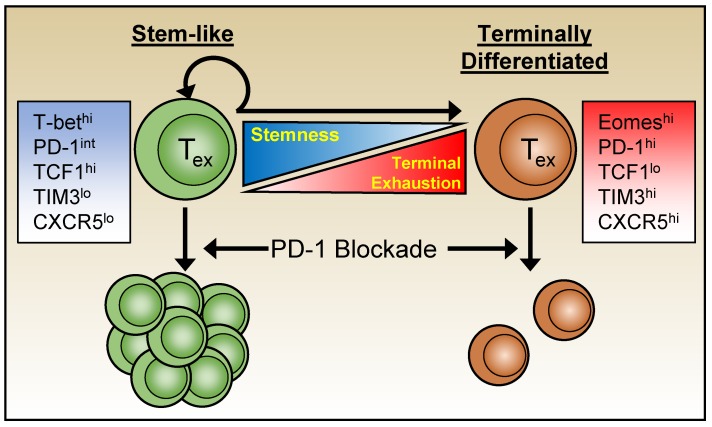
The exhausted CD8 T cell pool is heterogeneous, containing both populations of terminally exhausted cells (right) that are refractory to checkpoint blockade therapy, as well as populations of more “stem-like” cells (left) that can seed the terminally differentiated pool as well as persist and self-renew. This less terminally differentiated stem-like population is more capable of expansion following checkpoint blockade.

Variations in function, inhibitory receptor levels, transcriptome, and epigenetics segregate exhausted cells into heterogeneous subpopulations. Moreover, the use of checkpoint blockades, such as anti-PD-1 therapies have shown that not all exhausted cells can be rescued equally and permanently, prompting further characterization of exhausted subsets [48,96,99,107] (Figure 2). The search for subsets that are more amenable to immune therapy led to the identification of populations of exhausted CD8 T cells with a “stem-like” ability to self-renew and with greater developmental pluripotency. A subset of exhausted CD8 T cells that express high levels of T-bet and elevated but intermediate levels of PD-1 (T-bet^hi^ PD-1^int^) have been identified during chronic LCMV infection and shown to be more sensitive to PD-1 blockade therapies than Eomes^hi^ PD-1^hi^ subsets. Moreover, the Tbet^hi^ PD-1^int^ subset was discovered to be a progenitor population, seeding the more terminally exhausted Eomes^hi^ PD-1^hi^ population [99].

The transcription factor TCF1 (*tcf7*) is associated with stem-like properties of exhausted cells and their ability to self-renew. TCF1^hi^ CD8 T cells have greater proliferative capabilities, similar to the earlier identified Tbet^hi^ PD-1^int^ progenitor subset. These TCF1^hi^ progenitor cells also express CXCR5 and Bcl6, thereby sharing features with CD4 T-follicular helper cells (Tfh), and parallel CXCR5^+^ progenitor subsets identified in other studies [101]. While both exhausted CXCR5^+^ and CXCR5^-^ CD8 T cells express PD-1, the CXCR5^+^ population generally displays intermediate levels and is more sensitive to PD-1 blockade treatments [48,96,107]. Collectively, these studies further highlight the heterogeneity of exhausted T cells and demonstrate that collections of transcriptional regulators govern the complexity and composition of the exhausted pool. Most importantly, they influence the ability of these anti-viral T cells to persist over time and provide some level of viral control as well as calibrate sensitivity to checkpoint blockade therapies.

### 2.4. Epigenetics

**Figure 3:**
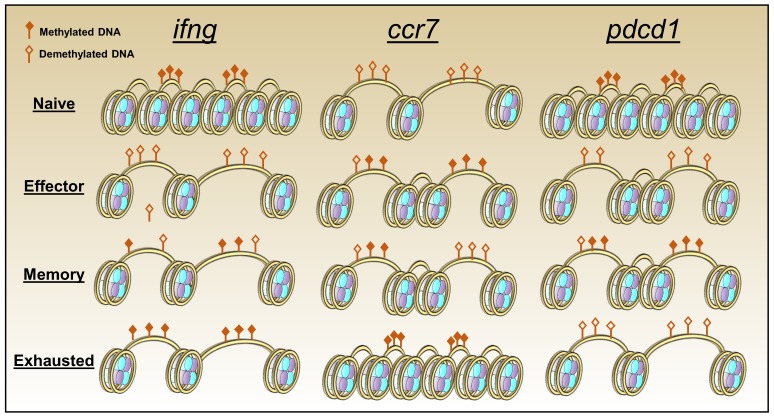
CD8 T cell exhaustion is characterized by a unique pattern of chromatin accessibility and DNA methylation. Over the course of CD8 T cell differentiation, effector associated *(ifn*g), memory-associated (*ccr7*) and exhaustion-associated genes (*pdcd1*) undergo unique shifts in chromatin accessibility which impact the availability of transcription factor binding sites and the ability of each gene to be transcribed. Notably, in exhausted cells the *ifn*g locus remains epigenetically open but undergoes de novo methylation. Conversely, the *pdcd1* locus remains demeythylated and actively expressed in exhausted CD8 T cells.

Upon activation T cells undergo significant epigenetic changes which help steer the development of effector and memory cells, and distinct patterns of modification are detected at each phase of differentiation. Notably, the epigenetic profile of exhausted CD8 T cells is distinct from that of conventional effector and memory cells. Furthermore, genomic analysis revealed that there are more epigenetic than transcriptional differences between virus-specific CD8 T cells during acute and chronic LCMV infections, indicating that epigenetic modifications may play a more significant role in sustaining exhaustion than transcriptional differences [91] (Figure 3). In exhausted CD8 T cells there is greater chromatin accessibility and DNA demethylation in the upstream region of the exhaustion-associated gene *pdcd1* which encodes PD-1. Conversely, the transcriptional permissiveness is diminished at memory associated gene loci such as *ccr7*, which can re-enforce the dysfunctional transition to memory characteristic of exhaustion. Transcriptional accessibility at the *ifn*g locus is also reduced, contributing to loss of T cell function [91,108].

Many of the epigenetic features of exhausted T cells are also permanently imprinted and resistant to reversal [109]. Elevated PD-1 expression and functional deficiencies are maintained following the adoptive transfer of exhausted LCMV-specific CD8 T cells [110,111]. The resilience of exhausted T cells to reversal of their epigenetic state is also apparent following PD-1 blockade [109]. This treatment temporarily enhances the transcription of effector-associated genes, cytokine production, and proliferation [109]. Analysis of the epigenetic profile of these virus-specific cells after anti-PD-1 blockade revealed that they maintain an epigenetic state associated with exhaustion despite their transient re-invigoration [109], and by 28 days after treatment, cytokine production and the transcriptional profile of the treated cells revert to again resemble that of their untreated counterparts.

Given this resistance to epigenetic change, the use of pharmacological epigenetic modifiers to reinvigorate exhausted T cells has become a logical direction to explore for developing therapies that can break this imprinting. The levels of diacetylated histone H3 become progressively reduced in exhausted CD8 T cells and this downregulation is associated with loss of functionality [112]. When exhausted CD8 T cells are treated with valproic acid, an inhibitor of histone deacetylase, to expand the degree of histone acetylation, there is an increase in IFN-γ and TNF-α production. Moreover, the conditional deletion of the DNA methyltransferase DNMT3a in activated CD8 T cells during chronic LCMV infection lead to the adoption of a T-bet^hi^ Eomes^lo^ stem-like phenotype and the virus-specific CD8 T cells were more amenable to PD-1 blockade therapies. This supports the concept that epigenetic modifications influence the formation of stem-like exhausted T cell subsets and dictate the efficacy of rejuvenation therapies [90]. Additionally, the use of the demethylating agent 5-aza-2’-deoxycytidine, in conjunction with PD-1 blockade, synergizes with and prolongs the benefits of PD-1 blockade [90]. These studies demonstrate that exhaustion is a durable state that is both inheritable as well as resistant to being rewritten by checkpoint blockade therapies. However, epigenetic modulators have the potential to reverse the epigenetic signatures of exhaustion and may have utility in bolstering immunity to persistent infections.

### 2.5. Metabolism

Cellular metabolism is critical for meeting the bioenergetic needs of the cell as well as for providing the substrates for epigenetic modifications including acetyl-coenzyme A for histone acetylation and S-adenosyl methionine for DNA methylation [113,114]. As naïve T cells become activated they shift their metabolism from mitochondria-based oxidative phosphorylation (OXPHOS) and enter glycolysis, which is less efficient but can quickly produce ATP necessary to support rapid proliferation and effector differentiation [115]. Following the peak of the effector response the surviving cells shift back to OXPHOS which sustains their long-term survival and the persistence of immunological memory. Curtailing glycolysis impedes effector formation and drives premature memory formation demonstrating that metabolism can dictate T cell fates, function and longevity [116]. Since both effector functions as well as memory development are corrupted during chronic LCMV infection understanding how glycolysis and OXPHOS affect exhaustion are critical questions.

During the initial stages of chronic LCMV infection the responding CD8 T cells show defects in their glycolytic pathways which are not apparent during acute infection and can impact the cell’s ability to clonally expand and attain effector activities [72,75]. These exhausted precursors are transcriptionally biased towards OXPHOS and have greater mitochondrial mass than cells from acutely infected hosts; however, T cells in the chronic environment have profound defects in their mitochondrial organization and respiratory capacity which impacts their long-term survival and contributes to the failure to establish memory [117,118,119]. These findings have led to investigations into whether redirecting the metabolism of exhausted CD8 T cells can influence their function.

**Figure 4:**
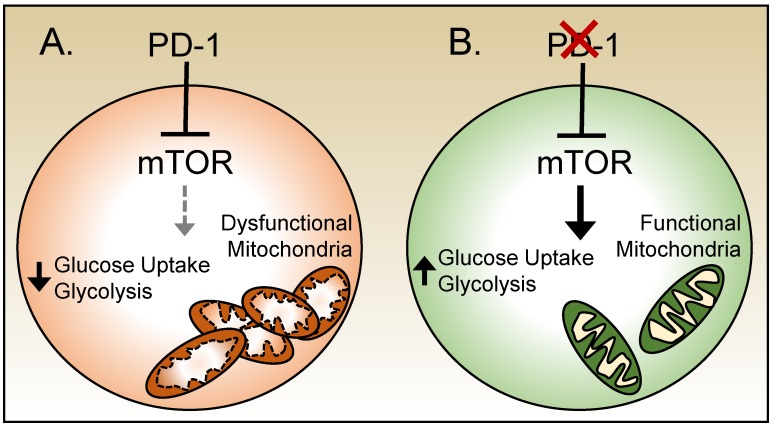
PD-1 and mTOR modulate metabolism in exhausted T cells. (**A**) During chronic viral infections PD-1 expression by exhausted CD8 T cells contributes to aberrant mTOR signals, which reduces glucose uptake and glycolysis, and also leads to an increased mass of disorganized and dysfunctional mitochondria. (**B**) Ablation of PD-1 enhances mTOR signals. This increases glucose uptake and glycolysis, as well as results in more organized and functional mitochondria.

PD-1 signals have been shown to inhibit the uptake of glucose and shift T cells toward OXPHOS [74] (Figure 4). PD-1-deficient CD8 T cells in mice chronically infected with LCMV maintained more organized mitochondria and exhibited greater glycolytic activity comparable to their counterparts generated in response to acute infection [72]. PD-1 has been shown to inhibit the functions of mTOR, a sensory molecule that is central to cellular metabolism [73,75]. mTOR functions are aberrant during chronic LCMV infection and in these circumstances persistent low levels of signaling through mTOR have been observed [72,75]. Inhibiting mTOR directly improved mitochondrial integrity but only mildly reduced glucose uptake and failed to improve effector functions during chronic LCMV infection. Transient treatment with anti-PD-1 antibodies increased signaling through mTOR and glucose uptake in CD8 T cells, demonstrating another potential mechanism by which checkpoint blockades can revitalize T cell functions [72,75] (Figure 4). However, co-treatment with anti-PD-1 antibodies along with rapamycin, an mTOR inhibitor, nullifies the benefits from the PD-1 blockade [75]. Together these data highlight the coupling between the exhausted phenotype and metabolic state which contributes to and maintains the ineffective response.

## 3. Extrinsic Drivers of T Cell Exhaustion

**Figure 5:**
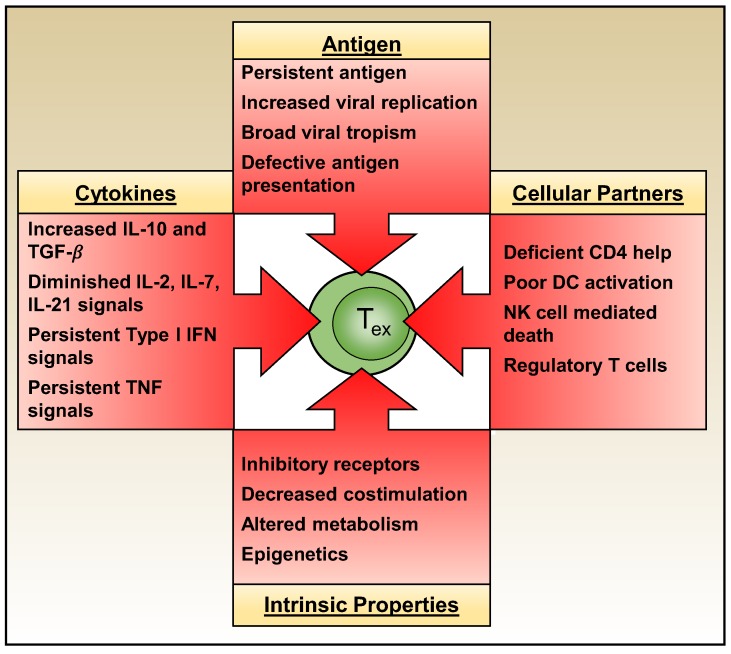
CD8 T cell exhaustion is regulated by the integrated actions of both extrinsic and intrinsic factors. Antigen availability and the cytokine milieu, together with other cellular partners all influence the development and maintenance of exhaustion. Exhausted T cells also self-regulate by their expression of inhibitory receptors and costimulatory molecules, as well as through their metabolic state and epigenetic landscape.

As discussed above, exhausted T cells attain unique intrinsic properties which cement and perpetuate their dysfunctional state. Whether T cell exhaustion develops, however, is governed by multiple extrinsic factors including the levels and duration of antigenic stimulation, the actions of other immune effector cells, and the composition of the cytokine milieu (Figure 5). 

### 3.1. Antigenic Signals

T cell responses are antigen-driven. During acute infections a relatively short exposure to presented viral-antigen is the primary signal that directs the elaboration of a robust effector response and the establishment of a permanent memory pool which helps to counter viral re-exposures. During the early stages of chronic LCMV infections the failure to contain the virus allows repetitive and prolonged episodes of antigenic stimulation which pushes the differentiation of exhausted T cells. During this initial phase the removal of CD8 T cells from the chronic antigenic environment leads to the recovery of effector functions and memory formation, demonstrating developmental plasticity as the cells first differentiate [110]. Similarly, if viral loads can be brought under control then some level of functional recovery may occur [38]. However, if an exhausted phenotype becomes firmly established then removal from chronic antigenic stimulation cannot rescue the virus-specific CD8 T cells [110,111,120].

Higher viral doses result in more severe exhaustion and CD8 T cells in tissues where viral antigen persists express higher amounts of inhibitory receptors and are less functional [57,121,122]. Consistent with the findings from LCMV, during HIV-1 infections correlations between viral levels and T cell functionality have also been reported. In these cases, the numbers of polyfunctional virus-specific CD8 T cells deteriorates but the levels of PD-1 expression are higher, further indicating the roles of antigen availability in determining the extent of exhaustion [60,83,123,124]. However, not all epitope specificities of LCMV-reactive CD8 T cells become equally exhausted [28,39,40,125]. This likely reflects the roles of both the absolute levels of presentation of individual viral epitopes as well as the types of cells that preferentially present these particular peptides in causing exhaustion [40,126]. 

Manipulating the extent of antigenic signals that CD8 T cells perceive also moderates the level of exhaustion in LCMV-specific CD8 T cells. Normalizing the overall amount of virus at the onset of the response while reducing the amount of available LCMV GP33 epitope curtails exhaustion of the GP33-specific CD8 T cell population [127]. Moreover, if viral antigen presentation is restricted by limiting the expression of MHC I to only hematopoietic or dendritic cells then exhaustion is also reduced [128,129]. Gradually increasing the numbers of MHC I expressing cells intensifies exhaustion in a dose dependent manner [129]. Conversely, when viral antigen is abundantly presented by non-hematopoietic cells then virus-specific CD8 T cells eventually become exhausted [129]. Additionally, the anti-viral CD8 T cell response can exert selective pressure on the virus, especially during the early stages of the infection. Consequently, the in vivo selection of escape mutants within the GP33 epitope of LCMV Clone 13 has been shown to limit the inactivation of the GP33-specific response while other viral epitope-specific populations of CD8 T cells still succumb to exhaustion [58]. Thus, viral antigen plays a critical and dynamic role is shaping the emergence and extent of CD8 T cell exhaustion.

Antigenic signals not only play a vital role in promoting exhaustion but may also be necessary for the continued survival of dysfunctional T cells. Exhausted CD8 T cells are less sensitive to IL-7 and IL-15 which usually promote the homeostatic maintenance of conventional memory T cells. Instead, exhausted CD8 T cells are more dependent on antigenic signals than cytokines for their persistence and these cells are unable to survive over time upon transfer into recipient hosts that do not express their cognate antigen [120,130]. 

### 3.2. Cellular Partners

#### 3.2.1. Ineffective CD4 T Cell Help

CD4 T cells play a key role in priming and preserving immune responses during both acute and chronic viral infections. While the requirement for CD4 T cell help varies depending upon the pathogen, their necessity is clear-cut during chronic LCMV infections [28,38,131,132,133]. The absence of CD4 T cells during the onset or throughout the infection leads to severe CD8 T cell exhaustion, poor humoral immunity, and high levels of viral persistence. Moreover, the co-transfer of CD4 and CD8 T cells into mice persistently infected with LCMV enhances the retention of functional CD8 T cells and the drop in viral titers when compared to the provision of CD8 T cells alone [134]. Exhausted CD8 T cells can also be rescued during chronic LCMV infection by the transfer of naïve virus-specific CD4 T cells. This boosts both the numbers and functional efficacy of virus-specific CD8 T cells in addition to improving the B cell response and lowering viral levels [135].

Arguably, the primary role for CD4 T cells during chronic LCMV infection is to support the CD8 T cell response and limit the severity of exhaustion. This is achieved by the ability of the CD4 T cells to provide cytokines including IL-2 and IL-21, in addition to their capacity to promote dendritic cell licensing through CD40:CD40L interactions. This provision of co-stimulatory signals and cytokines and chemokines fosters the recruitment and priming of the T and B cell response [136,137,138,139,140,141].

Like their CD8 counterparts, CD4 T cells also undergo exhaustion during chronic LCMV infection [38,142,143,144]. However, the process of CD4 T cell exhaustion is far less well-defined. Exhausted CD4 and CD8 T cells share several properties: they lose the ability to synthesize cytokines, including IL-2, TNF-α, and IFN-γ; they upregulate PD-1; they fail to differentiate into conventional memory subsets; and they mount less robust recall responses [142,143]. However, exhausted CD4 T cells are also distinct from their CD8 counterparts as they express lower levels of LAG-3 but higher levels of CTLA4, ICOS, and the transcription factor Helios.

Exhausted CD4 T cells are also distinct from other CD4 T cell subsets, but during chronic LCMV infections they share certain properties with Tfh cells, including the expression of Bcl6 and CXCR5 [145,146]. The proportion of exhausted CD4 T cells with this pseudo-Tfh phenotype increases over time during chronic LCMV infection, and the targeted deletion of these cells compromises the late arising, high affinity, neutralizing anti-viral antibody response which aids containment of the infection [145,147]. Interestingly, IL-6, which controls Tfh differentiation, is expressed in a biphasic manner during chronic LCMV infection, and inhibiting this cytokine reduces Tfh numbers and increases viremia [148]. Thus, in addition to directly helping the anti-viral CD8 T cell response, CD4 T cells likely assist the formation of a B cell response that further contributes to the control of the chronic infection. 

Tregs also expand during chronic infections, including LCMV, and influence the development of exhaustion [86,149,150]. Tregs can suppress responses by a variety of mechanisms including by impairing APC functions, competing for antigen, and consuming IL-2, as well as by producing immunosuppressive cytokines including IL-10 and TGF-β [151]. During chronic LCMV infection the administration of IL-2 increases the fraction of Tregs leading to a decrease in the abundance and functionality of the anti-viral CD8 population and an increase in viral levels [150]. Conversely, the removal of Tregs has been shown to bolster the anti-viral CD8 T cell response, limiting exhaustion, without affecting viral loads [86]. However, viral loads are reduced when Treg depletion is combined with PD-1 blockade, indicating that inhibition of the immune response by Tregs plays a complimentary role with other immunosuppressive mechanisms to maintain exhaustion and viral persistence. Interestingly, although the CD4 T cell derived cytokine IL-21 has been shown to act directly on anti-viral CD8 T cells to limit exhaustion, it also suppresses the Treg response, which further curtails the functional inactivation of the CD8 T cell response [150]. Together, these findings demonstrate the complex and multi-faceted roles of CD4 T cells in steering exhaustion during chronic viral infections.

#### 3.2.2. Natural Killer (NK) Cells

Natural killer (NK) cells are innate lymphoid cells that play substantial roles in the initial control of certain viral infections by mediating direct killing of infected cells, by secreting anti-viral cytokines, and by causing antibody-dependent cellular cytotoxicity [152]. Despite this classical role in host defense, the actions of NK cells encourage exhaustion during the early stages of chronic LCMV infection [153,154,155,156]. This is brought about by their ability to cull CD4 T cells thereby depriving the struggling CD8 T cell response of vital help. NK cells can also dampen the response by directly targeting CD8 T cells as well as producing suppressive cytokines such as IL-10 [152]. This NK cell-dependent moderation of the response pushes exhaustion and leads to viral persistence. Nevertheless, this appears to be critical for curtailing otherwise lethal immunopathology resulting from a toxic combination of a more disseminated viral infection together with a more aggressive T cell response [155]. 

The NK cell response does not completely ablate the anti-viral T cell response and the ability of the responding T cells to resist NK cell attack is governed by type I interferons (IFN-I). The receipt of IFN-I signals by activated T cells causes upregulation of the non-classical MHC-I molecule Qa-1b and limits expression of the NK cell activating receptor NCR1 [157,158]. In this way, IFN signals act to protect the activated anti-viral T cells from disposal by NK cells, and allows at least some level of response to materialize. Nevertheless, while IFN-I may play a supportive role in preventing NK cell-mediated killing of T cells, sustained IFN-I signals can amplify exhaustion.

### 3.3. Cytokines

#### 3.3.1. Proinflammatory Cytokines

Although the production of IFN-I is typically a major innate antiviral defense mechanism, which helps control the infection and augments immunity, these usual protective functions are disrupted during chronic infections [159]. Sustained elevated levels of IFN-I have been detected during several chronic viral infections including LCMV, SIV, and HIV [20,160,161,162]. IFN-I blockade treatments prior to or during chronic LCMV infection reduces T cell exhaustion and improves viral control [20,161]. When the roles of IFN- α and IFN- β were separately evaluated, IFN-α blockade altered viral dissemination but failed to lower viral loads [163]. By contrast, IFN-β blockade reduced viral levels, maintained lymphoid architecture, and preserved the T cell response, demonstrating the distinct roles of IFN-I molecules during chronic infections. Mechanistically, IFN-I signals promote the synthesis of IL-10 and expression of PD-L1 by dendritic cells resulting in an immunosuppressive signature [164]. IFN-I may also impede the anti-viral T cell response by promoting the terminal differentiation of exhausted cells at the expense of forming TCF-1^+^ CXCR5^+^ CD8 T cells which have greater proliferative and self-renewal capabilities and are more sensitive to reinvigoration by checkpoint blockades [101]. Additionally, cAMP responsive element modulator (CREM) levels in CD4 T cells are reduced upon IFN-I blockade [165]. CREM represses IL-2 expression by modifying chromatin accessibility of the *il2* locus [166]. Since the loss of IL-2 production is an early feature of exhausted T cells it is plausible that chromatin remodeling due to IFN-I signaling is one mechanism that advances and sustains this state.

In contrast with these studies, the early treatment of chronically infected mice with IFN-I rescued the CD8 T cell response and resulted in viral control [167]. This illustrates the dynamic role of IFN-I during chronic LCMV infection, with early production beneficial for viral control, but persistent exposure restricting the response which further drives exhaustion. This disfavoring of the anti-viral response may reflect an alternative protective role for IFN-I. In this case, rather than promoting immunity and viral clearance it instead acts to prevent immunopathology.

In addition to IFN-I, other proinflammatory cytokines also influence the progression of exhaustion, viral clearance, and the differentiation of immune cells during chronic viral infections. During chronic LCMV infection IL-6 expression peaks 1–3 days after infection and again around day 25 [148]. The late IL-6 response was shown to be critical for supporting the CD4 Tfh response and containing viremia [148]. Both IL-6 and the related cytokine IL-27 have been shown to support IL-21 production by CD4 T cells and viral control [168]. However, their precise roles apparently bifurcate, with IL-6 playing a more focused role in expanding the Tfh response and IL-27 supporting the overall survival of virus-specific CD4 T cell pool during the chronic infection [168].

Tumor necrosis factor (TNF) is another proinflammatory cytokine linked to immune exhaustion under certain conditions. Analysis of exhausted CD4 T cells during HIV-1 infection revealed that TNF signals induce several exhaustion associated pathways, including PD-1 expression [169]. The importance of TNF was probed using an early in life LCMV infection model where TNF levels become elevated, and a chronic infection ensues. In this system neutralization of TNF restored virus-specific T cell numbers, decreased levels of PD-1, increased cytokine production and reduced viral loads. However, TNF levels are not universally elevated during all chronic infections and remain low even in certain persistent LCMV infection models. Thus, the broader utility of this approach is not clear but it may be useful in a restricted manner for example during chronic HIV infections where increased levels of TNF are present.

#### 3.3.2. Immunosuppression by IL-10 and TGF-β

Immunosuppressive cytokines act to quell the immune response to limit immune pathology and induce tolerance; however, during chronic infections they can also suppress viral clearance and encourage exhaustion [170]. IL-10 levels become elevated during numerous chronic infections including LCMV as well as Epstein-Barr Virus (EBV), HIV, HBV, and HCV [171,172,173,174,175,176,177,178]. During chronic LCMV infections the cellular sources of IL-10 shift with dendritic cells and macrophages accounting for the majority of early production and CD4 T cells becoming the predominant later source [179], and these distinct cell types may make temporarily distinct contributions to facilitating T cell exhaustion. At the transcriptional level, IL-10 production by CD4 T cells is dependent on Blimp-1 which is highly expressed by exhausted T cells.

IL-10 has a wide array of immunosuppressive actions including stifling T cell proliferation, cytokine production, antigen presentation, and costimulation by APCs [180]. Genetic deletion or blockade of IL-10 during chronic LCMV infection curtails exhaustion and supports viral control [172,174,177]. However, the success of IL-10 blockade during chronic LCMV infection is dependent on the virus and timing of the treatment, both of which influences the severity of exhaustion [172,174,181]. IL-10 blockade also synergizes with PD-1 inhibition to more robustly reverse exhaustion and control viral loads [181,182]. However, while IL-10 producing CD4 T cells contribute to immune exhaustion, during chronic LCMV infection a distinct population of IL-10 producing CD4 T cells also develops that displays a Tfh phenotype and helps to sustain the humoral immune response to promote viral control [183], highlighting the need to further explore how different producers of IL-10 independently guide exhaustion.

Increased expression of the immunosuppressive cytokine TGF-β has been reported during several chronic viral infections including LCMV. This cytokine acts to modulate cell proliferation, survival, and differentiation [184]. During chronic LCMV infection the expression of the TGF-β receptor, TGF-βRII, is elevated in virus-specific T cells and the levels of phosphorylated SMADs, which are induced by TGF-β signaling, are increased [185,186]. TGF-β acts on CD4 T cells in chronically infected mice, inhibiting their proliferation and differentiation, potentially further contributing to global exhaustion [187]. The use of a dominant-negative TGF-βRII system, which ablates signaling, limited the exhaustion of anti-viral CD8 T cells and improved viral control [185,186]. The interpretation of the role of TGF-β during chronic LCMV infection is, however, complicated by enhanced endogenous T cell activation which manifests in mice expressing the dominant negative TGF-βRII. Moreover, blocking TGF-β only marginally improves T cell functions and fails to impact viral control [185,188]. Thus, the precise contributions of TGF-β to T cell exhaustion have not been fully deciphered.

#### 3.3.3. Common-Gamma Chain Receptor Family Cytokines: IL-2, IL-7, IL-15, and IL-21

IL-2, IL-7, IL-15, and IL-21 are members of the common gamma-chain receptor family of cytokines that function to steer T cell responses and provide critical support for the differentiation, proliferation, and homeostatic maintenance of naïve, effector, and memory T cells. During chronic LCMV infection T cells rapidly lose the ability to produce IL-2. Treatments that boost IL-2 levels during chronic LCMV infection enhance the number of virus-specific CD8 T cells and reduce viral loads [87,189,190]. However, the administration of IL-2 also expands the Treg population in chronically infected mice, which can offset benefits of cytokine therapy and further stifle the immune response [150]. This implies that IL-2 therapy needs to be carefully calibrated to guide differentiation and steer development away from the production of Tregs and toward more favorable T cell responses.

Interestingly, during chronic LCMV infection CD8 T cells genetically engineered to lack expression of the high affinity IL-2 receptor CD25 are not maintained over time, suggesting that IL-2 signals are either required to sustain the exhausted cells or are necessary at some point to imprint the self-renewal and survival traits of certain exhausted subsets [189]. Exhausted cells have been shown to arise from KLRG-1^lo^ effector-like precursors which are not strong manufacturers of IL-2 [110]. Thus, IL-2 may play a critical time-dependent role in the formation and survival of exhausted cells. CD122 (IL-2Rβ), a receptor shared by IL-2 and IL-15, becomes upregulated as exhaustion develops which correlates with the emergence of impaired functionality and increased PD-1 expression. Deletion of CD122 lessens exhaustion and increases functionality, implying that the integration of IL-2 and IL-15 signals influences the ontogeny and fates of exhausted CD8 T cells [191]. Although the expression of CD122 is prolonged on LCMV-specific CD8 T cells during chronic LCMV infection when compared to parallel populations in acutely infects hosts, expression on exhausted cells does eventually subside [59,191].

IL-7 plays a pivotal role in the homeostasis and survival of naïve and memory T cells. Chronic LCMV infection suppresses the expression of the IL-7 receptor CD127 which reduces T cell survival and memory formation [192,193]. IL-7 therapy in mice chronically infected with LCMV bolsters the numbers of functional anti-viral T cells and decreases the amount of virus. This viral control is directly driven by T cells since their elimination negates the benefits of IL-7 therapy [22,23]. Treatment with exogenous IL-7 also decreases the levels of suppressor of cytokine signaling 3 (SOCS3) which restrains the anti-viral immune response during chronic LCMV infection. This, in turn, leads to increased levels of IL-6, a cytokine shown to help support the helper and humoral immune response [23,148]. However, since the progression and severity of exhaustion sways the levels of IL-7 receptor expression on virus-specific T cells, the success of IL-7 therapy depends on timing and is more successful if administered during the early contraction phase. PD-1 checkpoint blockade therapies have been shown, however, to elevate the expression of CD127 on exhausted cells, thus increasing their sensitivity to IL-7 [109]. This provides a therapeutic opportunity for IL-7 administration, which can synergize with PD-1 blockade to help restore T cell numbers and the functions of exhausted cells.

During chronic LCMV infection CD4 T cells are primary producers of IL-21 [194,195,196]. IL-21 or IL-21 receptor deficiency leads to severe exhaustion and the inability to control chronic LCMV infection. IL-21 acts directly to preserve anti-viral CD8 T cells via STAT3 dependent signals and interactions with the transcriptional regulators IRF-4 and BATF, and CD8 T cells which lack expression of IL-21R are rapidly lost during chronic LCMV infection [194,195,196]. Conversely, IL-21 treatments within the first week of infection augment viral clearance and improve anti-viral CD8 T cell functions but result in lethal disease [196]. IL-21 not only directly regulates the CD8 T cell response but also limits Treg development and helps humoral responses [150]. The ability of IL-21 to contain the induction of Tregs during chronic LCMV infection also likely indirectly contributes to the preservation of the anti-viral T cell responses, as well as permitting more effective antibody production [197]. In the absence of IL-21 signals, an early humoral response is initiated but it is not sustained over time [198] and elimination of a subset of CD4 T cells that produce IL-21 and IL-10 during chronic LCMV infection leads to the impairment of the virus-specific antibody production [183]. Thus, common gamma chain receptor family cytokines play distinct and multifaceted roles in determining whether T cell exhaustion develops and persists.

## 4. Conclusions

Lymphocytic choriomeningitis virus was instrumental in the discovery of T cell exhaustion over 20 years ago. Building on this initial breakthrough has tremendously advanced our understanding of the complexity and heterogeneity of cellular immunity. Importantly, the findings from LCMV have helped to guide broader advances and we now appreciate that different degrees of T cell dysfunctionality and exhaustion commonly manifest during numerous persistent infections as well as during tumor outgrowth. Importantly, a better understanding of exhaustion has prompted the design of strategies to prevent and reverse this condition with the goal of improving viral clearance and especially tumor control. New technologies have allowed a more precise definition of the transcriptional, epigenetic and metabolic features of exhausted T cells, and have helped reveal the heterogeneity of exhaustion. These insights have enhanced our ability to predict the populations that respond to treatments and which therapeutic combinations successfully synergize. Nevertheless, important gaps in our understanding remain. It is unclear how the level and degree of heterogeneity within the exhausted pool becomes established and maintained; how molecular regulators of exhaustion integrate to dictate cell fates has not been well deciphered; CD4 T cell exhaustion remains poorly understood; and the full impact of exhaustion on the overall immune competency of the host is not clear. Given the utility of the LCMV system it is likely that it will remain at the forefront of exhaustion research and help resolve these and other unanswered issues.
